# Molecular Events in Primary and Metastatic Colorectal Carcinoma: A Review

**DOI:** 10.1155/2012/597497

**Published:** 2012-05-09

**Authors:** Rani Kanthan, Jenna-Lynn Senger, Selliah Chandra Kanthan

**Affiliations:** ^1^Department of Pathology and Laboratory Medicine, University of Saskatchewan, Saskatoon, SK, Canada S7N 0W8; ^2^Royal University Hospital, Room 2868 G-Wing, 103 Hospital Drive, Saskatoon, SK, Canada S7N 0W8; ^3^Department of Surgery, University of Saskatchewan, Saskatoon, SK, Canada S7N 0W8

## Abstract

Colorectal cancer (CRC) is a heterogeneous disease, developing through a multipathway sequence of events guided by clonal selections. Pathways included in the development of CRC may be broadly categorized into (a) genomic instability, including chromosomal instability (CIN), microsatellite instability (MSI), and CpG island methylator phenotype (CIMP), (b) genomic mutations including suppression of tumour suppressor genes and activation of tumour oncogenes, (c) microRNA, and (d) epigenetic changes. As cancer becomes more advanced, invasion and metastases are facilitated through the epithelial-mesenchymal transition (EMT), with additional genetic alterations. Despite ongoing identification of genetic and epigenetic markers and the understanding of alternative pathways involved in the development and progression of this disease, CRC remains the second highest cause of malignancy-related mortality in Canada. The molecular events that underlie the tumorigenesis of primary and metastatic colorectal carcinoma are detailed in this manuscript.

## 1. Introduction

Despite increased general awareness, colorectal cancer (CRC) remains the second leading cause of cancer-related death in Canadian men and women combined [1], with a third of CRC patients dying from this disease [2]. These are grim statistics given that this cancer is a well-studied malignancy with defined risk factors, a slow progression, and preneoplastic lesions that can be detected and treated by colonoscopic polypectomy [3]. Though 5-year survival rates for early stage cancers (Dukes A and B) is up to 95% and 60–80% respectively, survival rates drop dramatically to 35% with lymph node involvement (Dukes C), indicating early detection and treatment is imperative for best patient management [4].

Recognition that histologically identical tumours may have drastically different prognosis and/or response to treatment prompted the theory that, rather than a single malignancy, CRC is a heterogenous, multifactorial disease [5, 6]. It is theorized, perhaps, that individual tumours are initiated and progress in a unique manner that is not necessarily identical amongst all tumours [7]. As a result, the focus of CRC research is shifting from a clinical perspective towards developing an understanding of the molecular basis of this malignancy, including individual susceptibility, development, progression, response, and resistance to antitumour treatment and metastatic spread [8]. Cancer develops through multiple and sequential genetic alterations [3, 9], and some patients may have synchronous alterations in two or three different pathways [10]. Through clonal selections, the cancer cell “chooses” the genetic alterations most conducive to growth through proliferation of cells that possess the desired qualities with apoptosis of those that do not [2]. A more thorough understanding of these molecular pathways may contribute to improved strategies for prevention, screening, diagnosis, and therapy. The following is an overview of the molecular events in primary and metastatic CRC.

## 2. Materials and Methods

 A detailed review was conducted in the published English literature limited to the past five years (since 2006). The search was performed using PubMed and Google Scholar with the text phrases “molecular pathway” and “colorectal.” Articles were read, analyzed, and screened with a focus on colorectal cancer/carcinoma. Primary reference lists from these manuscripts as well as PubMed's “Related Articles” feature were used to identify additional relevant articles.

The aim of this review is to discuss molecular changes occurring in

- primary CRC, including genomic instability, genomic modifications, microRNA, epigenetic changes,
- metastatic CRC (mCRC) including growth factors and epithelial-mesenchymal transitions (EMTs).

## 3. Genomic Instability

 The first model of colorectal tumorigenesis put forward by Fearon and Vogelstein outlined a four-step sequential pathway for the development of cancer, in which

Step  1: *APC* inactivation causes adenoma development,
Step  2: *KRAS* mutations promote adenomatous growth,
Step  3: genetic alterations of chromosome 18q allowed progression with biallelic loss,
Step  4: p53 inactivation triggers the final transition to carcinoma [11, 12].

Recent insights suggest this pathway, now over twenty years old, requires refinement to include new findings [13]. Further, this sequence is thought to occur in only 60% of cases [14]. In the process of elucidating the true pathogenesis of CRC, controversy has emerged between those postulating that genomic instability is necessary to elucidate the multiple mutations present in CRC and those disagreeing, hypothesizing instead that cells continuously produce genetic changes with those that confer a survival advantage being selected through clonal expansion [12].

Only 5% of adenomas will progress to cancer development, indicating that carcinogenesis requires additional molecular modifications, with greater emphasis on increased proliferation [15, 16]. It is suggested CRC is initiated by “cancer-initiating stem cells” with the ability to self-renew, perpetuate, and generate a variety of differentiated cells. These cells are proposed to harbour the initial mutation of *APC* in one of the 10^7^ crypts of the gastrointestinal tract which then colonize the crypt with mutated cells [13].

Carcinogenesis is now viewed as an imbalance between mutation development and cell-cycle control mechanisms. When the cell-cycle is no longer capable of controlling the mutation rate, it is referred to as “genomic instability.” Three separate pathways have been identified that contribute to this imbalance:

1. chromosomal instability (CIN),
2. microsatellite instability (MSI),
3. CpG island methylator phenotype (CIMP).

These groupings are created in order to facilitate prediction of (a) patient prognosis, (b) response/resistance to therapies, and (c) possible etiological factors to optimize prevention [7].

### 3.1. Chromosomal Instability

 The chromosomal instability (CIN) pathway, also known as the suppressor pathway, is the most common type of genomic instability [8], encompassing 50–85% of CRCs [5, 17]. This pathway is characterized by karyotypic variability resulting from gains and/or losses of whole/portions of chromosomes [13]. Various mechanisms that contribute to CIN have been identified and categorized to include (a) sequence changes, (b) chromosome number alterations, (c) chromosome rearrangements, and (d) gene amplification [18]. Additional changes identified include chromosomal segregation defects/microtubule dysfunction, abnormal centrosome number, telomere dysfunction/telomerase overexpression, DNA damage, and loss of heterozygosity (LOH) [13, 18, 19]. Chromosomes that attach improperly to the mitotic spindle confer a higher risk of mis-segregation. This may be due to *APC* mutations, which normally interact with microtubule-binding-protein EB-1 to assure proper chromosomal attachment [20]. Telomerase dysfunction occurs when cells with shortened telomeres do not undergo apoptosis, leading to breakage-fusion-bridge cycles that can lead to genomic reorganization [13].

A common finding in chromosomally instable neoplasms is loss of 18q, a finding identified in up to 70% of primary CRCs [13]. Genes on this chromosome include *deleted in colorectal carcinoma (DCC)*, *SMAD2*, and *SMAD4*. The product of *DCC* is a cell-surface receptor for neuronal protein netrin-1 and is important in cell adhesion and apoptosis [5]. Mutations to this gene are rare (6% CRCs) [13]. *SMAD2 *and *SMAD4 *function in the TGF-*β*-signalling pathway (Section 4.1.3) [3]. 18q loss of heterozygosity (LOH) has been correlated with poor prognosis; however, the extent of the prognostic value requires further validation [5]. The “two-hit hypothesis” for LOH states that both alleles of a tumour suppressor gene must be inactivated in order to contribute to tumourigenesis. Often, this is accomplished by loss of one allele with inactivation by a point mutation of the other [21].

CIN can be detected early in a dysplastic crypt foci [3] and is more commonly found in the distal colon; however, whether or not it precedes *APC* inactivation remains unclear. Detection of these genomic changes often includes cytometry, karyotyping, LOH analysis, fluorescent *in situ* hybridization (FISH), and comparative genomic hybridization (CGH) [13]. This pathway results in a change in both the chromosomal copy number and structure [8] and are characterized by aneuploidy, transformations, and LOH that contribute to the inactivation of tumour suppressor genes such as *APC, DCC, SMAD4*, and *TP53* [4, 17, 22]. CIN has additionally been associated with loss of chromosome 5q, 17p, and 18q [3]. Though aneuploidy is recognized as occurring in tumours with CIN, the mechanisms responsible for this remain unknown. Therefore, some authors propose that cancer development through genomic instability may arise without mutations, simply through self-propagation. Cancers with aneuploidy often show mitotic abnormalities including centrosome numbers, multipolar spindles, and lagging chromosomes suggesting mitotic spindle checkpoint dysregulation [18].

 There remains no clear evidence to discern whether CIN is a cause or a consequence of malignancy [13, 20]. It is clear, however, that CRC with CIN confers a poor survival regardless of ethnicity, tumour location, and treatment with 5-FU [23]. The majority of CIN tumours are predominantly of the distal colon/left sided tumours present clinically with routine lower gastrointestinal symptoms that include bleeding per rectum and presence of a mass with or without obstruction. Histomorphologically, these lesions are either polypoid exophytic or indurated/ulcerated growths with the characteristic histology of moderately well-differentiated adenocarcinoma with a typical “dirty” necrosis of CRC.

### 3.2. Microsatellite Instability

 Microsatellite instability (MSI) is detected in 15% of CRCs and arises when microsatellites become abnormally long or short due to gain/loss of repeated units [24]. This pathway of genomic instability was first reported in 1993 at the detection of thousands of somatic alterations in a length of DNA from a CRC [25]. A microsatellite is a stretch of DNA containing a pattern of 1–5 nucleotides in length with tandem repeats [22, 24]. Microsatellites are found abundantly throughout the genome and are unique in uniform and length within a tissue of one individual [12]. A minimum of 500,000 microsatellites are estimated within the genome, occurring in the intron, untranslated terminal regions, and the coding exon itself [26]. Microsatellites may be classified as monomorphic (the same number of repeats in all individuals) or polymorphic (varied number of repeats among individuals) [26]. Elongation or shortening of the microsatellite is primarily due to inactivation of DNA mismatch repair (MMR) genes, which are responsible for correcting base-base DNA replication errors. At regions of short repeats within the genome, such as satellites, DNA polymerase is particularly susceptible to making mistakes; therefore, when MMR is inactivated and cannot correct these mistakes, MSI is the result [3]. This inactivation may be genetic or acquired. These tumours usually are not associated with mutations in *KRAS* or *TP53*; however, genes such as TGF*β*RII, EGFR, and BAX, which contain simple repeats, are often mutated in these tumours [5]. Additional genes affected by MSI include regulators of proliferation (GRB1, TCF-4, WISP3, activin receptor-2 insulin-like growth factor-2 receptor, axin-2, CDX), the cell cycle or apoptosis (caspase-5, RIZ, BCL-10, PTEN, hG4-1, FAS) and DNA repair (MBD-4, BLM, CHK1, MLH3, RAD50, MSH3, MSH6) [12].

MMR inactivation may be due to either an inherited germline mutation to one allele with somatic inactivation of the other or somatic inactivation of both alleles [26]. The most common mechanism of MMR inactivation is through an acquired methylation of the h*MLH1* gene promoter [17]. The MMR system comprises seven proteins (MLH1, MLH3, MSH2, MSH3, MSH6, PMS1, PMS2) which associate and form functional heterodimers [3]. These mutations allow repeats within the microsatellite to accumulate or to be lost through clonal propagation [27]. Standard sequencing for the detection of MSI can miss large deletions/rearrangements that arise in up to 1/3 of MMR mutations [24]. The most common detection mechanism is by length measurement of a PCR amplicon containing the microsatellite. Methylation-specific PCR to test for methylation is another simple test, where loss of h*MLH1* staining by immunohistochemistry correlates with MSI [26]. Though MSI is primarily due to MMR inactivation, some CRCs with intact MMR can develop MSI through frameshift mutations at microsatellites. A standardized panel for MSI testing includes two mononucleotides (BAT25 and BAT26) and three dinucleotide microsatellites (D5S346, D2S123, D17S250) [3]. The MSI expanded panel includes BAT40, myb, TGF*β*RII, IGF2R, and BAX for a 10-marker panel [27]. Based on these markers, the degree of MSI can be categorized as high MSI (MSI-H) when two or more panel markers are involved, low MSI (MSI-L) if only one marker is involved, and MSS if none. The value of a MSI-L category remains questionable, as it is argued if enough microsatellites in CRC are tested, eventually some instability will be detected [26]. The clinical presentation of MSI-L tumours has yet to be fully determined and this will likely result in acceptance or rejection of this category.

Clinicopathological features defining MSI-positive tumours are not reported consistently in the literature [28]. Most of these tumours usually arise from adenomas, are located proximal to the splenic flexure, and confer a better prognosis for their stage than microsatellite-stable (MSS) tumours [10, 17, 29–31]. Additionally, MSI is more common in females and arise in both younger and older population, though this has not been consistent in all studies. Patients can also present with synchronous and metachronous malignancies [28]. On microscopic examination, these tumours are often poorly differentiated with a mucinous phenotype and are associated with prominent intratumoral and peritumoral lymphocytic infiltration [4]. They often have a lower overall stage of disease but demonstrate deeper invasion [28]. Unlike CIN, these tumours do not have chromosomal abnormalities or allelic loss [4]. Many MSI-H tumours have a Crohn's-like inflammatory response near the tumour edge; however, no universal prognostic pathologic feature has been identified in all MSI-positive CRCs. Distant metastases are less common in MSI-positive CRC [28]. It remains uncertain if the favourable prognosis associated with MSI tumours is intrinsic or rather due to a greater sensitivity to chemotherapy. Adjuvant chemotherapy with 5-FU is not beneficial to MSI-H tumours as they demonstrate an altered response to both chemotherapy and radiotherapy. The use of MSI as a prognostic factor remains uncertain although it is suggested that it may be useful in the selection of individual therapeutic regimes [5].

Hereditary nonpolypsis colorectal cancer, now referred to as Lynch syndrome because of its extraintestinal manifestations, is due to a mutant germline MMR gene causing loss of MMR function with somatic inactivation of the wild-type allele [8]. These patients usually develop multiple tumours between 20–30 years of age in the colon and may have extracolonic manifestations in the rectum, endometrium, renal pelvis, ureter, stomach, ovary, skin, brain, and/or small intestine [12, 32]. It is estimated that 80% of Lynch syndrome carriers remain undetected in the population [25]. This syndrome makes up only 3% of CRCs [24] but is the most common inherited cause of CRC [26]. Germline mutations of *MLH1, MSH2, MSH6*, and *PMS2* have been associated with the development of this hereditary syndrome [32]. The majority of cases are due to autosomal-dominant inheritance of a mutation in either *MLH1 *or* MSH2.* Approximately 1/3 of these patients will have no pathogenic mutation in their mismatch repair genes [33]. In these cases, Lynch syndrome arises from germline epimutations inactivating genes through promoter methylation and is usually identified in families that show no MMR gene sequence mutation [12]. This most commonly occurs through hypermethylation at *EPCAM *that inactivates the downstream gene *MSH2 *[24]. To confirm the diagnosis, testing for BRAF is indicated, as it is virtually never mutated in Lynch syndrome [26] though it is mutated in 40–50% of sporadic MSI tumours [24].

### 3.3. CpG Island Methylation Phenotype

 The newest of the three genomic instability pathways, the CpG island methylator phenotype (CIMP) was originally grouped together with MSI. CpG islands are regions within the genome rich in CpG (Cytosine-phosphate-guanine) dinucleotides where cytosine DNA methylation does not covalently modify [2]. These islands are especially common in promoter sequences, found in over half of them [2]. In normal tissue cytosine methylation is common outside of the exons [8]. Methylation naturally increases with age, injury, and in patients with chronic inflammation [12].

 The epigenetic modification of methylation is well recognized as a crucial event in altering gene expression associated with carcinogenesis and is more frequent in cancer than genetic changes [5]. Methylation of promoter CpG islands occurs in all tissue types in carcinogenesis [34]. Methylation leads to transcriptional silencing of genes involved in tumour suppression, cell cycle control, DNA repair, apoptosis, and invasion [35]. CIMP positivity is found in 35–40% of CRCs and has additionally been identified in adenomas [17]. It is postulated DNA methylation may be altered in normal colorectal mucosa, predisposing the affected tissue to further dysplastic changes. It is the second most common cause of sporadic CRC [3]. Through hypermethylation of histone CpG islands, the chromatin closes into a compact structure such that the gene promoter is inaccessible to transcription factors, thereby inactivating gene transcription. Widespread hypermethylation resulting in greater gene inactivation is characteristics of a CIMP-positive tumour [5]. Tumour suppressors that are frequently inactivated in this epigenetic process include p16, p14, MGMT, and hMLH1 [17].

 Clinically, CIMP+ tumours share some characteristics with MSI-H tumours including a proximal location and a poor degree of differentiation. Unlike MSI, however, these tumours may have a particularly poor prognosis [17]. These tumours often have KRAS and/or BRAF mutations [5]. CIMP and CIN positivity are mutually exclusive [17]. Traditionally, CIMP was detected by FISH and LOH analysis studying only selected regions; however, with advances in microarray technologies, a genome-wide, high-resolution scan can be achieved with CGH and SNP arrays [21, 36].

 Similar to MSI, the prognostic significance of CIMP remains ill defined, with some studies finding an adverse effect on prognosis and others no effect [5]. As such CIMP+ tumours confer a worse prognosis than MSI tumours, though concurrent MSI with CIMP positivity may improve prognosis compared with an MSI−/CIMP+ tumour. It has been suggested the adverse effect associated with CIMP positivity may not be innate, but rather due to *KRAS* or *BRAF* mutations [5].

### 3.4. Alternative Serrated Neoplastic Pathway

In recent years, it has been recognized that besides the traditional adenoma-carcinoma sequence of CRC, approximately 35% of carcinomas arise from the serrated pathway, developing from precursor lesions often referred to as the “serrated polyp” [31]. Serrated polyps are lesions composed of epithelial infoldings creating a serrated appearance. Though there is currently no universally accepted nomenclature, these lesions include typical hyperplastic polyps (HPs), sessile serrated adenomas (SSAs), and dysplastic serrated polyps (SSADs). There is ongoing nomenclature wars between groups that hold sessile serrated adenoma as a misnomer as they lack cytological dysplasia but harbour architectural crypt disorder and demonstrate disordered proliferation in contrast to traditional “hyperplastic polyps” versus “sessile serrated polyp” versus “sessile serrated lesion” [29]. Two types of hyperplastic polyps are recognized both by image enhanced endoscopy and histology: (a) goblet cell serrated polyps (GCSPs) and (b) microvesicular serrated polyps (MVSPs). These are also genetically dissimilar as GCSPs are associated with KRAS mutations while MVSPs are linked with BRAF mutations with increased susceptibility to aberrant methylation at the CpG rich island (CIMP) [29]. Dysplastic serrated polyps include (a) sessile serrated adenomas with dysplasia (SSAD), (b) traditional serrated adenomas (TSAs), and (c) conventional adenomas with serrated architecture. It is currently believed that SSADs have a greater risk to progress to MSI-H colorectal cancers. This serrated neoplastic pathway of colorectal carcinogenesis is usually found in females with an average age of 61 years and arise predominantly from precursor MVSPs which differ from traditional hyperplastic polyps as they have crypt architectural alterations that reflect disordered growth with dysmaturation. On endoscopy, serrated polyps may be overlooked as they are often flat or sessile. These lesions are suspected to arise more often in the proximal colon, are more common in females, and generally arise a decade later than the average CRC age [14]. Approximately 20% of CRCs originate from the serrated pathway of neoplasia. In this context, two separate molecular pathways have been proposed.

- BRAF-mut with CIMP-H is seen in the majority of syndromic, nonsyndromic cancers, and MSI cancers. 12–15% of MSI cancers occur by epigenetic silencing of the promoter methylation of DNA mismatch repair gene hMLH-1 as the key step leading to MSI with rapid progression from low to high grade dysplasia to invasive cancer.
- KRAS-mut are CIMP-low, no hMLH-1 activation, and are MSS with many of them harbouring p53 mutations like conventional CRCs. CIMP-high cancers are seen in the proximal colon, in females, have prominent glandular serrations with mucinous differentiation or poorly differentiated glands (medullary/undifferentiated) with intratumoral lymphocytes and Crohn's-like nodular peritumoral infiltrates.

Currently, no management guidelines for serrated polyps have been formalized [29–31]. The risk of metachronous and synchronous neoplasia in patients with serrated polyps is also not clearly defined; however, most evidence points to large serrated polyps being considered as a marker for synchronous advanced colorectal neoplasia and certain proximal or dysplastic serrated polyps increase the risk of metachronous serrated and/or conventional adenomas. Further, in the context of “interval cancers,” these are 2.5x more likely to demonstrate CIMP+, 2.7x more likely to demonstrate MSI and nearly 2x more likely to occur in the proximal colon; thus, MSI, and CIMP were independently associated with interval cancers [37]. It is currently believed that many of these interval cancers originate from the serrated neoplastic pathway outlined above.

 Various combinations of these pathways exist. Four molecular subtypes of CRCs and their precursor lesions are identified on the basis of both CIN and MSI statuses: (i/ii) conventional adenomas can give rise to CIMP−/MSI− (75–80% CRCs) or CIMP−/MSI+ (5% CRCs) tumours, (iii) sessile serrated adenomas can give rise to CIMP+/MSI+ (10% CRCs) tumours, and (iv) serrated adenomas (sessile or traditional) can give rise to CIMP+/MSI− (5–10% CRCs). Of these combinations, CIMP+/MSI− tumours confer the worst outcome with metastases being most common in these tumours, in 43%, followed by 18% in CIMP−/MSI−, 6% in CIMP−/MSI+, and none in CIMP+/MSI+ in Kang's study. The clinical outcome for MSI-negative tumours worsens when correlated with methylation. In tumours positive for MSI, those also positive for CIMP conferred a worse prognosis [38]. Chromosomal instability and microsatellite instability are mutually exclusive [17]. Recognition of genomic instability and the subtype is important to guide systemic therapy and affects outcome [23].

## 4. Genomic Modifications

### 4.1. Mutational Inactivation of Tumour-Suppressor Genes

 Tumour suppressor genes code proteins that act to limit growth and proliferation, the cell cycle, motility, and invasion in normal human tissues [8]. In carcinogenic transformation, tumour growth is often facilitated by inactivation of these genes through deletions, mutations, promoter methylation, or mutation of one allele with loss of the other [12, 39]. Several key players in the carcinogenetic process have been identified and are well elucidated in the literature. These genes include *APC, TP53, *and *TGF-*β*.*

#### 4.1.1. *APC*

 The *APC* gene codes for the APC protein, a large structure that is involved in the regulation of differentiation, adhesion, polarity, migration, development, apoptosis, and chromosomal segregation [13]. The main action of the APC protein is within the Wnt signalling pathway. The canonical Wnt signalling cascade is a well-studied pathway suspected to play an integral role in the development of cancer. When Wnt proteins bind to and activate the cell-surface receptors, these Frizzled proteins activate Dishevelled family proteins, which inhibits the “destruction complex” that includes Axin, glycogen-synthase kinase-3*β*, and APC. As such, the *β*-catenin within the cytoplasm will translocate to the nucleus where it acts as a cofactor for T-cell factor/lymphoid enhancing factor (TCF/LEF) transcription factors and regulates a wide variety of specific cells. Normally, Wnt ligand binding is absent, inhibiting the destruction complex, thus allowing it to carry out its action: the phosphorylation of *β*-catenin for ubiquitination and proteolytic degradation [2, 40].

Mutations of the *APC *gene result in a protein unable to induce *β*-catenin phosphorylation. Cytoplasmic *β*-catenin levels increase and migrate to the nucleus [40]. *APC* mutations, therefore, indirectly induce genes targeted by the TCF/LEF transcription factors including the proto-oncogene c-myc and cyclin D1 and genes encoding membrane factors (MMP-7, CD44), growth factors, and Wnt pathway feedback regulators [2, 22]. Such mutations have been detected in 5% of dysplastic aberrant crypt foci, 30–70% of sporadic adenomas, and up to 72% of sporadic tumours [13]. The altered APC protein is most commonly due to premature truncation during protein synthesis, in 95% due to frameshift or nonsense mutations [2]. Alternatively, *β*-catenin gain-of-function mutations with a fully intact *APC* gene have been detected in up to 50% of colonic tumours with the same result of increased proto-oncogene expression [13]. A mutation to either the *APC* gene or *β*-catenin that activates this signalling pathway has been detected in the majority of CRCs [40] and is suspected to be an initiating event of carcinogenesis [8]. This is substantiated by the finding that *APC* mutation is sufficient to cause growth of small benign tumours [41]. It has been suggested *APC *mutations may be a rate-limiting event in the development of most adenomas, and both alleles are often inactivated by this point [2].

The distribution of *β*-catenin within the cell once the *APC* gene has been mutated appears to be heterogeneous. Moderately well-differentiated adenocarcinomas tend to accumulate nuclear *β*-catenin at their invasive front as well as scattered in the nearby stroma; however, in the central differentiated areas, the *β*-catenin is detected on the cellular membrane without translocation. It is postulated the tumour microenvironment may be an important factor in CRC growth and dissemination. Growth factors, chemokines, inflammatory factors, and the extracellular matrix are suspected to interact with the Wnt-signalling pathway resulting in this heterogenous intracellular *β*-catenin distribution [40]. Recent studies indicate that some sessile serrated adenomas (SSAs) have aberrant *β*-catenin labelling implicating the Wnt pathway in the molecular progression of SSA to colorectal cancer. In the study by Yachida et al., abnormal *β*-catenin nuclear labelling is seen as a common features in serrated polyps with neoplastic potential and this correlates with early neoplastic progression following BRAF activation [42].

 The *APC* gene has been implicated in the development of familial adenomatous polyposis (FAP), an inherited condition characterized by hundreds to thousands of adenomas lining the large intestine by the second to third decade of life with a high propensity for malignant transformation early in life, between the ages of 40 and 50 [33]. This genetic disease accounts for less than 1% of CRC cases [9]. Patients with FAP carry a germline mutation of *APC* [8] which is autosomally dominant and is associated with almost 100% penetrance [43]. Extraintestinal symptoms include osteoma, dental abnormalities, congenital hypertrophy of retinal pigment epithelium, and extracolonic tumours [9]. Gardner's syndrome is also caused by *APC *mutations, and Turcot syndrome is suspected to be due to *APC* gene mutation or mismatch repair gene mutations [33].

#### 4.1.2. *TP53*

 The tumour suppressor protein p53 and its gene *TP53* is a well-studied element of the carcinogenic pathway, with alterations to its function found in most human cancers. The *TP53 *gene is found on the short arm of chromosome 17 and is induced by oncogenic proteins such as c-myc, RAS, and adenovirus E1A [13]. Normally, the p53 protein is negatively regulated by MDM2, E3-ubiquitin ligase, and MDM4 that targets p53 for ubiquination. In the presence of cellular stress, MDM2 and MDM4 have disrupted interactions with activation and transcription of p53 [13]. P53 protein then regulates cell cycle by activating DNA repair when necessary, arresting cells in the G1/S and the G2/M boundary when genetic damage is detected, and initiating apoptosis if the damage cannot be repaired [2]. Thus, p53, often designated as the guardian of the genome, has a key role in maintaining genomic stability.

 Inactivation of *TP53* is a key step in the development of CRC [8]. Mutations and LOH of p53 are important with the transition from adenoma to carcinoma [2]. Usually both alleles are inactivated by a missense mutation inactivating transcription and a 17p chromosomal deletion of the second *TP53* allele [8]. This loss of function is found in 4–26% of adenomas, 50% of adenomas with invasive foci, and 50–75% of CRCs [13]. The developing neoplasm places a variety of stresses on the cell, including DNA strand breakage, telomere erosion, hypoxia, reduced nutrient exposure, antiangiogenesis, and cell-cycle arrest. Ineffective p53 function prevents the protein from responding appropriately to these stimuli, thereby facilitating growth and invasion [2].

#### 4.1.3. TGF-*β*

 SMAD is the pathway through which the transforming growth factor beta (TGF-*β*) protein signals activity. The pathway is initiated by a TGF-*β* dimer binding to a TGF-*β* Receptor II that recruits and phosphorylates a type I receptor. This receptor then phosphorylates receptor-regulated SMAD (R-SMAD). The family of R-SMADs includes SMAD1, SMAD2, SMAD3, SMAD5, and SMAD8. This R-SMAD will then bind to SMAD4 to form a complex that enters the nucleus and affects transcription [2]. There, it causes apoptosis and cell cycle regulation.

 Approximately one third of CRCs demonstrate somatic mutations inactivating the *TGFBR2 *gene. Inactivating mutations of the TGF-*β* pathway is involved in the adenoma transition to high-grade dysplasia or invasive carcinoma [8].

*SMAD2 *and *SMAD4* encode the proteins SMAD2 and SMAD4 and are mutated in 5% and 10–15% of CRCs respectively [2]. *SMAD4* germline mutations are implicated in juvenile polyposis syndrome, an autosomal dominant condition in which multiple hamartomas develop throughout the gastrointestinal tract [44].

### 4.2. Activation of Oncogene Pathways

 Oncogenes are genes with the potential to cause cancer. They encode growth factors, growth factor receptors, signalling molecules, regulators of the cell cycle, and additional factors implicated in cellular proliferation and survival. When these genes are mutated, results may include overactive gene products, amplifications altering copy number, alterations that affect promoter function or modified interactions that cause transcription/epigenetic modifications.

#### 4.2.1. RAS and BRAF

 The RAS-RAF-MAPK pathway begins with a mitogen (such as EGF) binding to the membrane receptor (such as EGFR), which allows the GTPase Ras to exchange its GDP for a GTP, activating MAP3K (Raf) which activates MAP2K which activates MAPK. MAPK then activates transcription factors that express proteins with a role in cellular proliferation, differentiation, and survival [45].

 A key, and well-studied, component of this cascade is the Ras family, comprised of three members: KRAS, HRAS, and NRAS. These isoforms are located on the inner surface of the plasma membrane [45]. A common target of somatic mutations, especially at codons 12 (82–87%), 13 (13–18%), and 61, KRAS has been implicated in many human cancers [46]. KRAS mutations have been reported in 40% of CRCs and contribute to the development of colorectal adenomas and hyperplastic polyps [2]. These mutations are usually single nucleotide point mutations that lock the enzyme bound to ATP, by inhibiting its GTPase activities thus upregulating the Ras function [13, 22]. It, therefore, affects downstream signalling cascades including MAPK and PI3K.

 Early KRAS mutations have been identified in left-sided hyperplastic polyps [10]. This mutation is more common in polypoid lesions than nonpolypoid [6]. KRAS mutations are associated with a worse prognosis, in part due to the overexpression of KRAS contributing to metastases through increasing the production of protease to degrade the extracellular matrix. However, the prognostic role of KRAS mutations remains largely ill understood and further studies are required. Attempts have been made at targeting KRAS for cancer treatment including inhibition of protein expression through antisense oligonucleotides and with blockage of posttranslational modifications to inhibit downstream effectors [5].

KRAS mutations have recently gained interest as a negative predictive factor for anti-EGFR therapy response. Blocking EGFR will have no effect if KRAS is mutated as it functions downstream of EGF receptors. Thus, a KRAS mutation allows continual activation of the downstream pathway, thus negating the effects of the drug [41]. As such, anti-EGFR drugs (Section 7.1.2) are not recommended in KRAS-mutated tumours. In this context, it has been suggested that all patients with CRC under consideration for anti-EGFRs should be tested for KRAS mutation status prior to treatment initiation [16, 41].

 The Raf family includes three members: ARAF, RAF1, and BRAF. When activated, these serine/threonine kinases activate MEK1 and MEK2 which phosphorylate ERK1 and ERK2. The ERKs continue the cascade by phosphorylating cytosolic and nuclear substrates such as JUN and ELK1 that regulate a wide spectrum of enzymes such as cyclin D1 [13]. Similar to KRAS, BRAF mutations render it continually active, in over 80% of CRCs by substitution of thymine to adenine at nucleotide 1799 that results in a substitution of valine to glutamic acid [5]. These point mutations make BRAF an attractive marker for analysis, as they are present in at least 80% of mutants [22]. Such mutations are more frequent in MSI tumours and are reported in 5–15% of CRCs [5]. Mutations in BRAF and KRAS are mutually exclusive as they are intimately connected in the RAS-RAF-MAPK pathway [45]. In the rare instance of concomitant mutations, they confer a synergistic effect and increase tumour progression [5].

 BRAF mutations confer a worse clinical outcome and thus the need for adjuvant therapy [5]. Mutations are associated with a shorter progression free and overall survival [45]. Though controversial, some studies have found that these adverse clinical effects of BRAF are negated in CIMP+ tumours, suggesting the poor prognosis is not attributable to the BRAF mutation itself, but is probably attributable to the genetic pathway in which it occurs [5]. Similar to KRAS, BRAF mutations have also been implicated in anti-EGFR resistance. Approximately 60% of KRAS wild-type metastatic CRC (mCRC) are unresponsive to these drugs, and it is hypothesized that BRAF mutations may confer some of this resistance [41]. As such, BRAF mutation status may also be assessed to determine patients resistant to anti-EGFR therapy.

#### 4.2.2. Phosphatidylinositol 3-Kinase (PI3K)

The PI3K-Akt begins with activation of PI3K, which can occur in three ways: (1) a growth factor binds to IGF-1 receptor in the cell membrane, causing dimerization and autophosphorylation of the receptor, IRS-1 then binds to the receptor and acts as a binding site and activator of PI3K; (2) a growth factor binds to a receptor tyrosine kinase embedded in the membrane, again causing dimerization and autophosphorylation, the PI3K then binds directly to the receptor and is activated; (3) the GTPase Ras (as seen above) may bind PI3K and activates it. Once PI3K is activated, it detaches and phosphorylates PIP2 that is a component of the membrane, transforming it to PIP3. PIP3 then activates Akt, a proto-oncogene with functions including growth promotion, proliferation, and apoptosis inhibition [5, 22]. The system is restored by PTEN, which dephosphorylates PIP3 and inhibits Akt signalling.

 PI3Ks are a family of enzymes divided into three classes. Of interest in CRC is the class 1A PI3Ks, which are composed of one catalytic subunit (either p110*α*, p110*β*, or p110*δ*) and one regulatory subunit (p85*α*, p85*β* or p86*γ*) [22]. The catalytic subunit p110*α*, which is encoded by the protein *PIK3CA*, has been of particular attention as it is believed to play a significant role in cancer progression. Its mutation has been detected in approximately a third of CRCs [8]. These gain-of-function mutations cause increased Akt signalling even without the presence of growth factors [45]. Clinically, the prognostic role of *PI3KCA* is under investigation, and it is suspected to confer a poor outcome [5]. It has also been suggested to play a role in resistance to anti-EGFR treatment [45].

 Due to its inhibitory effect on Akt, the phosphatase and tensin homolog (PTEN) acts as a tumour suppressor gene in this pathway. In CRC, the *PTEN* gene may be inactivated by somatic mutations, allelic loss or hypermethylation of the enhancer region [45]. Mutation of *PTEN* is a later event in carcinogenesis that is correlated with advanced and metastatic tumours. Though it is clear that *PTEN* mutations in stage II CRC is a poor prognostic marker, its role in other stages of CRC remains uncertain [5]. There remains a discrepancy as to the exact role of *PTEN* in anti-EGFR resistance [45].

## 5. MicroRNA

 MicroRNA (miRNA) is short RNA 8–25 nucleotides in length that binds to mRNA to control translation of complementary genes [47]. Over 1,000 miRNA sequences have been identified, each controlling hundreds of genes for a total of over 5,300 genes, 30% of the human genome [48]. These short RNAs play a regulatory role in development, cellular differentiation, proliferation, and apoptosis. In this context, miRNA dysregulation is suggested to play a role in carcinogenesis when their mRNA targets are tumour suppressor genes or oncogenes through silencing and overexpression respectively [47]. Half miRNAs are located at the breakpoints of chromosomes and, therefore, at a higher risk of dysregulation [48]. Whether or not the microenvironment directly affects miRNA dysregulation remains unclear [49]. Mature miRNA conducive to tumour growth may be upregulated through transcriptional activation and/or amplification of the miRNA encoding gene and those unfavourable to growth are silenced by deletion or epigenetic modifications [47]. Overexpression of miR-31, -183, -17-5, -18a, -20a, and -92 and underexpression of miR-143 and -145 are common in CRC [50]. It remains unclear, however, which miRNA changes are causative and which are a result of carcinogenesis [2]. Many of the pathways explained in this manuscript can be affected by miRNA including [47]

- APC: miR-135a, miR-135b cause decreased translation,
- PI3K: miR-126 stabilizes PI3K signal, is lost in CRC,
- PTEN: miR-21 is repressed,
- KRAS: miR-143 causes decreases expression,
- p53: miR-34a induces apoptosis, is decreased in 36% of primary CRCs, miR-192, miR-194-2, and miR-215 are involved in cell cycle arrest and are also downregulated in CRC [48],
- EMT: miR-200c overexpression causes inhibition of ZEB1 and induces MET in cells that previously underwent EMT [48].

miRNA has the same potential for identification as mRNA/proteins for the screening, diagnosis, and prognostic prediction of CRC. They appear in both the serum and plasma. Each type of human cancer has a distinct miRNA expression pattern [2]. It has been further proposed that the miRNA pattern could be indicative of prognosis and act as a molecular target for treatment [49].

## 6. Epigenetic Changes

Epigenetic changes are those that alter genetic expression without modifying the actual DNA. These changes are detected in approximately 40% of CRCs [19]. These factors are conveyed during cellular division. Epigenetic changes, as they relate to CRC, can be subclassified into two broad categories: (1) histone modification and (2) DNA methylation.

### 6.1. Histone Modification

 Histones are proteins that package cellular DNA into nucleosomes and play a role in gene regulation. Covalent modifications, including acetylation, methylation, phosphorylation, and ubiquitinylation of these proteins can cause dense inactive heterochromatin to open to euchromatin and vice versa. Such modifications are reversible, usually occurring at the N- and C-terminal regions [51]. Hypoacetylation and hypermethylation are characteristic of transcriptionally repressed chromatin regions. Mutations in histones are most common at lysine and arginine residues [33]. The mutually exclusive modifications that have been identified in CRC include deacetylation and methylation of lysine 9 in histone H3. If acetylation occurs at this position, the gene is expressed whereas if it is methylated, the gene is silenced. It is suggested that a universal marker for malignant transformation is the loss of monoacetylation from Lys 16 and trimethylation at Lys 20 on histone H4 [51].

### 6.2. DNA Methylation

 The process of hypermethylation of CpG islands is discussed above (Section 3.3); therefore, this section will discuss the process of global DNA hypomethylation, a process not as well understood. Over 40% of human DNA contains short interspersed transposable elements (SINEs) and long interspersed transposable elements (LINEs) that are normally methylated but become hypomethylated in CRC development [35]. Hypomethylation most commonly occurs at repetitive sequences such as satellites or pericentromeric regions [51]. This epigenetic change increases chromosomal susceptibility to breakage, thus creating genomic instability, reactivating retrotransposons that disrupt gene structure and function, or activating oncogenes such as the S100A4 metastasis-associated gene in CRC [51]. These changes are believed to occur early in carcinogenesis, as hypomethylation is detected in benign polyps but is not changed once they become malignant [35].

## 7. Metastatic Colorectal Cancer

 The final stage of CRC involves detachment from the primary cancer, migration, access to the blood/lymph, and development of a secondary tumour [40]. Each step in metastatic spread requires definitive genetic and epigenetic changes; however, the exact mechanisms underlying these changes remain largely unknown. The complex pathway that drives progression cannot be assessed with a single marker that can accurately predict growth progression and prognosis. This indicates that a greater understanding of the multiple pathways and the molecular markers involved is necessary [16]. Growth factors such as prostaglandin E2, EGF, and VEGF as well as molecular mediators of the epithelial-mesenchymal transition have been identified as potentiators of metastatic spread. Biological agents specifically targeting these markers have increased the median survival time of mCRC to 23.5 months. However, this is associated with toxicities, a complex management plan, and a hefty financial burden [46]. Approximately 50% of patients with CRC will die due to complications of metastases; therefore, early recognition of these molecular changes involved in this process with development of therapeutic strategies to combat these events is a high priority in mCRC treatment [48].

### 7.1. Growth Factor Pathways

#### 7.1.1. Prostaglandin and Cyclooxygenase-2

 Prostaglandin E2 is mainly produced and secreted by fibroblasts in the stroma and epithelial cells. Its signal is transduced through interactions of endoprostanoid receptors. Activation of these endoprostanoids initiates a cascade that activates EGFR and PI3K/Akt signalling pathways resulting in *β*-catenin translocation to the nucleus [52]. Signalling of this prostanoid thus plays an important role in the development of adenoma and is strongly associated with CRC through the regulation of proliferation, survival, migration, and invasion [13]. These high levels may be induced by inflammation or mitogen-associated upregulation of cyclooxygenase-2 (COX-2), the mediator of prostaglandin E2, or due to a loss of 15-prostaglandin dehydrogenase (PDGH), the rate-limiting enzyme catalyzing prostaglandin E2 breakdown. Loss of PDGH is high and is reported in up to 80% of colorectal adenomas and carcinomas [8]. COX-2 upregulation can be induced by growth factors, cytokines, inflammatory mediators, tumour promoters, and is overexpressed in ~43% of adenomas and 86% carcinomas [13]. COX-2 has been implicated in angiogenesis, as overexpression of this enzyme induces the production of proangiogenic factors including VEGF and fibroblast growth factor [13]. The tumorigenic effects of COX-2 overexpression may be inhibited by anti-EGFR therapy [53].

#### 7.1.2. Epidermal Growth Factor (EGF)

Epidermal growth factor receptor (EGFR), a member of the HER-erbB family of receptor tyrosine kinases, is a cell-surface receptor that binds epidermal growth factor, transforming growth factor *α* (TGF-*α*), amphiregulin, betacellulin, and epiregulin [54, 55]. When bound, EGFR changes its conformation to activate its tyrosine activity and mediates signalling through activation of the RAS-RAF-MAPK and PI3K signalling cascades [55]. It may also activate phospholipase-C, STAT (signal transducer and activators of transcription protein), and SRC/FAK [45]. Dysregulation of EGFR signalling can result at multiple points in this pathway including (a) at the receptor (EGFR mutation, copy number change, overexpression), (b) at the transduction regulators (constitutive activation of RAS, RAF, PI3K), and (c) by methylation/mutations in genes coding these proteins [55]. Downstream targets of EGFR form an interconnected network of phosphorylation reactions that activate transcription factors to elucidate effects including tumour proliferation, angiogenesis, and cell survival [45]. Pathways activated by this receptor have been linked to molecules such as VEGF and hypoxia inducible factor *α*, both with a well-described role in promoting angiogenesis. Additionally, EGFR activation promotes invasiveness and spread by activating serine protease, which aids in the degradation of the extracellular matrix [53]. Finally, it is an initiating event in the RAS-RAF-MAPK pathway and the PI3K-Akt pathway as described above with prominent roles in carcinogenesis. Overexpression of EGFR occurs in 65–70% of CRCs, and as would be suggested by its effects, it is more commonly seen in advanced stage tumours [5]. In this context, EGFR has been identified as an important therapeutic target in metastatic CRC (mCRC).

 Overexpression is associated with an advanced stage, a worse histological grade, and lymphovascular invasion. Especially in the setting of mCRC, EGFR expression is suggested to be a potential prognostic factor; however, its impact on survival remains controversial. Nevertheless, pharmacological inhibitors of EGFR have significantly benefited the CRC patient population through both monoclonal antibodies to interfere with receptor signalling and tyrosine kinase inhibitors to interfere with the catalytic activity of the cytoplasmic domain [5]. Two monoclonal antibody (cetuximab and panitumumab) and two tyrosine-kinase inhibitor (Geftinib and Erlotinic) drugs have been created. The higher the patient's EGFR overexpression, the better the response to anti-EGFR treatment.

 Cetuximab is a human-murine immunoglobuin (IgG)G1 mAb that inhibits EGFR by binding to its extracellular domain in both normal and tumour cells. This binding results in receptor internalization, inhibiting it from binding a ligand. The most common toxicity of monotherapy cetuximab is an acneiform rash in 88% of patients followed by a hypersensitivity reaction in 10% [54]. The drug can be taken alone or in combination with other chemotherapies to improve survival of chemorefractory CRC [4]. A longer progression-free survival has been demonstrated in patients with mCRC taking cetuximab combined with FOLFIRI (leuvocovorin, 5-FU, irinotecan) [56].

 Panitumumab is a **κ** IgG2 mAb that binds with high specificity and affinity to the extracellular domain of the EGFR in both normal and tumour cells, whereby it prevents the binding of ligands, dimerization, autophosphorylation, and signalling [54]. When used in combination with chemotherapy or radiotherapy for the treatment of recurrent or first-line mCRC or in an adjuvant setting, both panitumumab and cetuximab drugs act synergistically.

 Gefitinib is an orally administered anilinoquinazoline that acts through reversible inhibition of EGFR tyrosine kinase autophosphorylation, thereby inhibiting the downstream signalling [54]. The most common toxicity was diarrhoea in two thirds of patients and neutropenia in 60% [54]. It does not mix well with irinotecan-based chemotherapies, and studies have shown no objective response to this drug [54].

 Erlotinib is a quinazolinamine that also reversibly inhibits EGFR tyrosine kinase to prevent receptor autophosphorylation. It has shown no meaningful activity as a single agent treatment for mCRC; however, its effects seem more promising when combined with oxaliplatin and fluoropyrimidine [54].

 A common risk of these targeted therapies is resistance in patients who initially responded to monoclonal antibodies or tyrosine kinase treatments. The mechanism behind this resistance remains poorly understood [41]. As noted in Section 4.2.1, the presence of KRAS or BRAF mutations has been found to limit the activity of these anti-EGFR drugs. As such, screening of CRC patients for treatment suitability is suggested in order to avoid unnecessary exposure to the drugs and to reduce treatment costs.

#### 7.1.3. Vascular Endothelial Growth Factor

Angiogenesis, the development of new blood vessels from preexisting ones, is normally a vital process during development and wound healing; however, in carcinogenesis, angiogenesis is necessary to transport oxygen and nutrients into a growing neoplasm. These two processes differ in the balance between pro- and antiangiogenic signals. Proangiogenic factors include vascular endothelial growth factor (VEGF), fibroblast-growth factor (FGF), platelet-derived growth factors (PDGFs), insulin-like growth factor (IlGF), and transforming growth factor (TGF), and antiangiogenic factors include thrombospondin-1, angiostatin, and endostatin [54]. In carcinogenesis, the balance between these factors is lost, with proangiogenic factors predominating. In CRC, neovascularization is driven by hypoxia stimulating the production of angiogenic factors such as VEGF [48].

Vascular endothelial growth factor (VEGF) is a key component of this process in both normal and pathologic tissues, activating endothelial cell growth, migration, differentiation, and vascular permeability [56]. This family of angiogenic and lymphangiogenic factors includes five VEGF glycoproteins (A-E) and placental growth factors (PGFs) 1 and 2 [54]. These ligands bind to the VEGF receptor (VEGFR 1–3), a tyrosine kinase transmembrane protein, to activate pathways such as RAF/MEK, ERK, AKT, mTOR, and PI3K [56]. Specific glycoproteins bind to specific receptors with varying affinity. VEGFR1 is a receptor for VEGF-B and PGF with a role in hematopoiesis, endothelial progenitor recruitment, and growth factor induction. VEGFR2 is a receptor for VEGF-A and -F that increases microvascular permeability, proliferation of endothelial cells, migration, and invasion. Finally, VEGFR3 binds to VEGF-C and -D to mediate embryonic cardiovascular development [54]. High serum levels of VEGF are associated with a poor prognosis [56], and *VEGFR1* gene expression may be predictive of tumour recurrence [16].

 Based on the important role that angiogenesis and VEGF play in advanced CRCs, a monoclonal antibody therapy was created to inhibit this process. Bevacizumab works against all isoforms of VEGF-A that inhibit binding to its receptor, causing regression of microvessels and inhibiting the formation of new vessels [54]. The only toxicity reported associated with its use is hypertension that is manageable with pharmaceuticals [56]. Additionally, it is suggested that Bevacizumab impacts vascular flow, facilitating the increased delivery of chemotherapy to the tumour [57]. Bevacizumab may be combined with the FOLFOX (leucovorin, 5-FU, oxaliplatin) or FOLFIRI (leuvocovorin, 5-FU, irinotecan) treatment regimens [56]. This drug combined with chemotherapy increases survival of patients with mCRC when compared with chemotherapy or bevacizumab alone [4]. Three VEGF tyrosine kinase inhibitors have additionally been created: Vatalanib, Afibercept, and Sunitinib [54, 56]. To this point, no other anti-VEGFs have shown efficacy in the treatment of mCRC.

### 7.2. Epithelial-Mesenchymal Transition

 Epithelial-mesenchymal transition (EMT) is a proposed mechanism that facilitates invasion and metastases in which epithelial cells are changed into dedifferentiated mesenchymal cells characterized by decreased E-cadherin, loss of cell adhesion, and increased cell motility [58]. This transition is induced by transcriptional repressor zinc-finger E-box binding homeobox (ZEB1) [47]. ZEB1 causes repression of E-cadherin transcription and is triggered by TGF-*β* [48]. These cells lose their intercellular connections that are normally mediated by E-cadherin, facilitating association with the extracellular matrix (ECM) for an anchor, and thus propel forward [58]. In this context, ECM remodelling by proteinases such as the urokinase plasminogen activator cascade and matrix metalloproteinases is central to tumour growth, survival, invasiveness, and metastases [47]. EMT signalling additionally causes *β*-catenin to stabilize and translocate to the nucleus. The EMT is completed when the basement membrane is degraded and the mesenchymal cell is formed as evidenced by loss of *β*-catenin and E-cadherin membranous expression [59]. These mesenchymal cells are then free to metastasize to distant sites and through mesenchymal-epithelial transition establish colonies histopathologically similar to the primary tumour [59].

 This series of events relies on signals from the stroma including hepatocyte growth factor, EGF, placental-derived growth factor, and TGF-*β*. These signals cause activation of transcription factors to induce EMT, including Snail, Slug, ZEB1, Twist, Goosecoid, and FoxC2 [59]. Hepatocyte growth factor (HGF) promotes the transcriptional activity of *β*-catenin in a self-amplifying positive feedback loop to promote growth and invasion. EGF, TGF-*β*, and placental-derived growth factor promote phosphorylation of p68 which binds to *β*-catenin and inhibits its stabilization by GSK3*β* [40]. Downstream targets of *β*-catenin are pro-metastatic and include galectin-3 and Fascin, both of which are expressed at the invasive margin and confer a poor outcome [40].

 In CRC, tumour budding is considered analogous to EMT. Tumour budding is considered the histological mark of EMT and is defined as the presence of dedifferentiated single cells/small clusters at the invasive front of colorectal cancer [60]. This process occurs in 20–40% of CRCs [59]. Tumour budding occurs at the invasive front by a small aggregate of cells detaching and migrating through the stroma and is considered the initiation of invasion and metastases [58, 59]. This process is more common in MSS CRC, which may partially explain the poorer prognosis of MSS when compared with MSI [58]. The Wnt/*β*-catenin pathway as previously described, as well as the polypeptide subunit of laminin 5 (LAMC2) in the ECM, has been implicated in tumour budding initiation. Budding is independently associated with poor survival [58]. The presence of buds is predictive of metastases to the lymph nodes through the tumour vessels and lymphatics as well as distant metastases, and local recurrence [59]. A study by Zlobec et al. showed that in patients with a KRAS mutation, high-grade tumour budding is predictive of a nonresponse to anti-EGFR therapies with up to 80% accuracy [60].

## 8. Detection of Molecular Events

 Though colonoscopy remains the gold standard for CRC screening, fewer than 60% of those eligible over the age of 50 have undergone this test. Reasons for this may include procedural factors such as test discomfort, invasiveness, embarrassment, and lack of availability of trained personnel/facilities/equipment [61]. Further, despite the uncertainty, the malignant potential serrated lesions is beyond doubt as they represent the precursors to an important proportion of the overall CRC burden. Therefore, such lesions are also regarded as important targets for the development of CRC prevention strategies. As many of these lesions are sessile, and not easily identified on routine colonoscopy, there is a further increased interest in the development of other alternative modalities for the screening of CRC [31]. Blood and feces are the two media in which targets for earlier molecular detection of CRC have been developed.

### 8.1. Fecal

Fecal occult blood testing (FOBT) is a noninvasive method of testing for blood in the stool that is commonly conducted as a first-line screening for CRC. This screening tool has reduced CRC mortality by 15–33% [62, 63]. Guaiac-based FOBT detects hemoglobin's peroxidase activity. As it will react to the presence of blood from any site, it is not specific for colorectal bleeding and, therefore, false positives may result from upper gastrointestinal bleeding as well as nonhemoglobin sources of peroxidase such as drugs, vegetables, and red meat. As such, patients are asked to withdraw ASA and NSAIDs a week prior to the test, and fresh vegetables/meat 3 days prior. Compliance is, therefore, a problem, and small adenomas that normally do not bleed are often undetected. More recently, with the development of immunological FOBT that uses antibodies against human globin such dietary and medication restrictions are not required. Additionally, as the antibodies in this test are aimed at globin chains that would be degraded if the bleed initiated in the upper gastrointestinal tract, the sensitivity is improved [22].

There has been a recent interest in the detection of molecular markers in fecal specimens as a screening tool for CRC. This detection is based on the natural process of coprocytobiology (colonocytes shedding from the colonic mucosa and being excreted in the stool at a rate of 10^10^ cells/day) [64, 65]. Cells derived from neoplastic lesions are more resistant to destruction and can survive longer in the stool, preserving their genetic aberrations. For the detection of these molecular changes, the fecal samples are stored fresh in a DNA-stabilizing buffer that delays degradation for several days [66, 67]. Once in the laboratory, human genetic materials have to be separated from bacterial, as only 0.01% of the genetic material in feces is human [66]. Genetic material is then amplified with PCR after which genetic molecular markers can be detected through many different methodologies. A wide variety of genetic, epigenetic, and multimarker panels have been suggested as markers to indicate neoplastic growth including *KRAS*, *TP53*, *APC*, and *COX-2*. The role of fecal molecular markers in CRC screening has been recently reviewed from our laboratory and is, therefore, not further discussed in the current manuscript [68].

Stool testing confers a wide spectrum of advantages over flexible sigmoidoscopy/colonoscopy. As colonocytes are shed from the entire length of the colon, fecal genetic material detected is representative of the same; therefore, this screening tool is able to identify lesions undetectable by a flexible sigmoidoscopy that reaches only the distal third [69]. Additionally, patients are more likely to comply with this stool testing, as it is noninvasive, requires no dietary or medicinal restrictions [70], and does not necessitate an office visit [71]. One study found that 91% of patients indicated they would be willing to take the test again in the future [72]. The greatest challenge lies in the identification and selection of a cost-effective single or a select panel of molecular markers with high sensitivity and specificity for CRC.

### 8.2. Blood

 Tumour cells circulating in the peripheral blood may be reflective of tumour genetic characteristics and, therefore, useful in predicting metastatic and recurrence potential [73]. Blood tests are relatively non-invasive and more convenient for the patient than fecal screening. Additionally, blood generally does not contain microflora, reducing interference with genetic material thus facilitating laboratory analysis [73].

More than forty serum proteins have been identified as potential CRC biomarkers, as annotated in Creeden et al.'s recent review [73]. Many of these proteins are for early detection of cancer development. One of the oldest and well-researched markers is carcinoembryonic antigen (CEA) that is positive once a CRC is established; however, it does not readily detect early disease [22]. CEA also has a poor sensitivity and specificity and is clinically used to monitor progression and treatment response in CRC [74]. Studies analyzing methylated DNA detection in the serum/plasma are limited; however, promising results suggest a potential role in the future [74]. The primary challenge remains in the identification of a serum marker specific for colorectal adenoma and carcinoma, as many biomarkers being found are positive in other cancers or in nonneoplastic inflammatory diseases [73].

## 9. Conclusion

**Figure 1:**
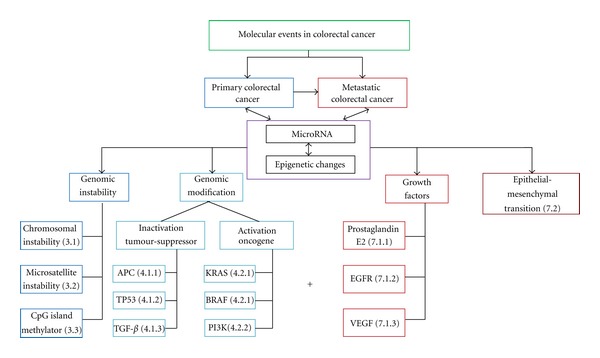
Molecular events in colorectal cancer. This is a pictorial representation of the molecular events in primary (blue) and metastatic (red) colorectal carcinoma. The + sign indicates additional molecular events that are recognized in the metastatic progression of this disease. The designated events are referenced to their numerical section within the manuscript.

 In conclusion, it is, therefore, apparent that, despite many strides in understanding the development and progression of CRC, a lot remains largely unknown due to the multifactorial process of this disease as summarized in Figure 1. Despite the abundance of molecular pathways and markers continually being reported, CRC mortality rates remain high, indicating poor translation from the laboratory to the clinical setting [16]. The goal for the future lies in the development of research tools that can target early detection of many of the ongoing well-recognized subcellular molecular events to improve the burden of mortality and morbidity related to colorectal carcinoma.
